# Experiences from patients in mental healthcare accessing their electronic health records: results from a cross-national survey in Estonia, Finland, Norway, and Sweden

**DOI:** 10.1186/s12888-024-05916-8

**Published:** 2024-07-02

**Authors:** A.J. Fagerlund, Annika Bärkås, A. Kharko, C.R. Blease, J. Hagström, I. Huvila, I. Hörhammer, B. Kane, E. Kristiansen, S. Kujala, J. Moll, H. Rexhepi, I. Scandurra, S. Simola, H. Soone, B. Wang, R.M. Åhlfeldt, M. Hägglund, M.A. Johansen

**Affiliations:** 1 Norwegian Centre for E-health Research, University Hospital of North Norway, Tromsø, Norway; 2 Participatory eHealth and Health Data Research Group, Department of Women’s and Children’s Health, Uppsala University, Uppsala, Sweden; 3 MedTech Science & Innovation Centre, Uppsala University Hospital, Dag Hammarskjölds väg 14b, 1 floor, Uppsala, 75185 Sweden; 4 Faculty of Health, University of Plymouth, Plymouth, UK; 5 Digital Psychiatry, Dept of Psychiatry, Beth Israel Deaconess Medical Center, Harvard Medical School, Boston, MA USA; 6 Department of ALM, Uppsala University, Uppsala, Sweden; 7 Department of Computer Science, Aalto University, Espoo, Finland; 8 Business School, Karlstad University, Karlstad, Sweden; 9 Centre for Health Policy and Management, Trinity College Dublin, Dublin, Ireland; 10 Centre for Empirical Research on Information systems, School of Business, Örebro University, Örebro, Sweden; 11 School of Informatics, University of Skövde, Skövde, Sweden; 12 E-Medicine Centre, Department of Health Technologies, Tallinn University of Technology, Tallinn, Estonia

**Keywords:** Patient-accessible electronic health records, PAEHR, Online record access, ORA, Electronic health records, EHRs, Mental healthcare

## Abstract

**Background:**

Patients’ online record access (ORA) enables patients to read and use their health data through online digital solutions. One such solution, patient-accessible electronic health records (PAEHRs) have been implemented in Estonia, Finland, Norway, and Sweden. While accumulated research has pointed to many potential benefits of ORA, its application in mental healthcare (MHC) continues to be contested. The present study aimed to describe MHC users’ overall experiences with national PAEHR services.

**Methods:**

The study analysed the MHC-part of the NORDeHEALTH 2022 Patient Survey, a large-scale multi-country survey. The survey consisted of 45 questions, including demographic variables and questions related to users’ experiences with ORA. We focused on the questions concerning positive experiences (benefits), negative experiences (errors, omissions, offence), and breaches of security and privacy. Participants were included in this analysis if they reported receiving mental healthcare within the past two years. Descriptive statistics were used to summarise data, and percentages were calculated on available data.

**Results:**

6,157 respondents were included. In line with previous research, almost half (45%) reported very positive experiences with ORA. A majority in each country also reported improved trust (at least 69%) and communication (at least 71%) with healthcare providers. One-third (29.5%) reported very negative experiences with ORA. In total, half of the respondents (47.9%) found errors and a third (35.5%) found omissions in their medical documentation. One-third (34.8%) of all respondents also reported being offended by the content. When errors or omissions were identified, about half (46.5%) reported that they took no action. There seems to be differences in how patients experience errors, omissions, and missing information between the countries. A small proportion reported instances where family or others demanded access to their records (3.1%), and about one in ten (10.7%) noted that unauthorised individuals had seen their health information.

**Conclusions:**

Overall, MHC patients reported more positive experiences than negative, but a large portion of respondents reported problems with the content of the PAEHR. Further research on best practice in implementation of ORA in MHC is therefore needed, to ensure that all patients may reap the benefits while limiting potential negative consequences.

**Supplementary Information:**

The online version contains supplementary material available at 10.1186/s12888-024-05916-8.

## Background

Patients’ online record access (ORA) enables them to read the content of their electronic health records (EHR) from any device with an internet connection. The patient-accessible electronic health record (PAEHR) allows reading of various health data, including clinical assessments, discharge letters, radiology and laboratory results, nursing documentation, as well as information about allergies and medication [1], depending on the set up in the particular country. The service is now available on a nationwide level in many countries through national health portals, and is reaching maturity. In some countries, patients’ ORA are emerging. For example, a recent policy in the United States requires healthcare institutions to provide patients with access to their EHRs, in a format that can be exported to third-party applications [2].

In Estonia, the nationwide health information system that includes a patient portal called *Digilugu* has been operating since the end of 2008 [3]. Since 2010, all residents with electronic ID have been able to see their PAEHRs, from both primary and secondary care, including epicrisis, lab and examination results, diagnosis and prescriptions. Over the years an expanded range of medical documents has been added in a step-by-step manner [4]. In Finland, the implementation of the national patient portal *My Kanta* started in May 2010, and access is offered to all citizens with a Finnish personal identity number [5]. *My Kanta* offers patient ORA with access to clinical notes and laboratory results from all authorised Finnish healthcare providers, including primary and secondary care, public and private healthcare, and social welfare providers [6]. In Sweden, PAEHR implementation started in 2012 when the national PAEHR service *Journalen* was made accessible to all residents with electronic ID in the Uppsala region. Since then, the PAEHR service has spread to other regions and by the end of 2018 all 21 regions in Sweden had implemented *Journalen* [7]. Geographically, there are differences in PAEHR implementation within Sweden, since the regions use different technical solutions for EHRs and enforce different policies for information access and information sharing. Hence, even though all Swedish residents have access to *Journalen*, the type of information that is accessible differs depending on where patients receive care. All regions show clinical notes and diagnoses from somatic care, and 17 of the 21 regions give patients access to their records from psychiatric care [8]. Records from both primary and secondary care can be accessed. In Norway, PAEHR implementation started in 2015, and in 2019 three out of four health regions provided their patients with the PAEHR service *Pasientsjournal* [9], while the final region implemented the service after this study was conducted. There are also regional differences in Norway in which elements in the medical record are made available and to whom. In the Northern Norway health region, for instance, records from both somatic and mental healthcare (MHC) are treated equally with full access, while in other regions they are differentiated, i.e. perhaps a different time of implementation or limitations in mental healthcare. Furthermore, health records from general practitioners are not accessible through PAEHR in Norway. While there are no uniform statistics in the Nordic countries specifically on PAEHR usage, national usage statistics from patient portals, hubs for several services including PAEHR, indicate that these portals are widely adopted within the populations (34).

The evolution of ORA in Nordic countries has its roots in the ‘Scandinavian Approach to participatory design’ [10], This approach embraces democratic principles and patient/consumer empowerment, and inclusivity, with respect to technologies including how they are designed and implemented. In contrast, in April 2021, the move to embrace ORA in the US was motivated by the 21st Century Cures Act [11] with the goal of moving towards a health app economy, with the aim of accelerating medical product development and bringing innovations to patients faster.

Results from previous research indicate that ORA for patients with (MHC experience may strengthen patient autonomy [12], contribute to patient-centred care [13], improve adherence to medications [14], and enhance patient empowerment [15, 16]. Interestingly, some results suggest that ORA may increase trust between healthcare personnel (HCP) and patients in groups that may be at a disadvantage in the healthcare system such as older individuals and non-white patients [17], and Sámi patients in Norway [18]. A recent scoping review summarised that while ORA in MHC may benefit patients, HCPs overall express concerns outweigh the benefits [19]. Indeed, the implementation of PAEHRs, especially in MHC has been the subject of debate. For example, in Sweden, HCPs raised multiple concerns in response to the decision that mental health information should be made accessible in *Journalen*. Before the implementation in Skåne, the first region making MHC records accessible to patients, HCPs expressed concerns regarding patient behaviour after access; and expressed an intention to document more restrictively with psychiatric records accessible online, along with a fear of increased tension between HCPs and patients, and worry about threats and violence from patients [20, 21]. Similar findings emerged in the Uppsala region [22]. In Norway, HCPs in MHC raised concerns that patients might get angry or upset from reading their notes, or have their condition deteriorate after using PAEHR during an unstable episode, and suggested that the service might not be suitable for everyone [23, 24], thus raising the question whether there should be limitations for patient ORA. Furthermore, recent unpublished results from Norway suggest that patients that received MHC at more severe levels of care such as emergency and inpatient care have more negative experiences with PAEHR [25], and in Sweden, patients in MHC identified more errors in their documentation compared to non MHC-patients [26].

The legality of limiting access to PAEHR on a diagnostic basis has been questioned by legal professionals in Norway, with claims that decisions that limit access must be based solely on individual rather than group considerations [27]. In 2015, in the Northern Norway health region that implemented PAEHR in somatic healthcare and MHC simultaneously, there were more concerns among HCPs for MHC than somatic shortly after implementation, with HCPs in MHC reporting that the ORA leads them to document differently [24], for example by being more careful in their wording or trying to avoid provoking the reader [9]. The tendency to worry more about MHC was slightly reduced, but still present three years after the implementation, and several of the MHC providers kept shadow records on their own computer, or on paper [28].

Currently, there are few studies exploring users’ experiences with ORA in MHC [19]. Some small studies suggest that patient ORA can both enhance trust and undermine it; trust appeared to be compromised if patients felt offended, surprised, or if their notes were incongruent with what was discussed in clinic visits [29]. A secondary analysis of a large survey of patient access to clinical notes in the US found that patients with serious mental health diagnosis were more likely to report understanding their medications including side effects, feel more in control, and to report doing a better job taking their medications as prescribed after online access [14]. In a qualitative study among the Norwegian Sámi minority, MHC patients reported that ORA enabled them to check the therapist’s understanding and perceptions of the discussion that took place during the consultations [18].

An international Delphi study [30] of 70 mental health professionals, patients, and researchers drawn from six countries where PAEHR is implemented in MHC also revealed mixed findings with panellists agreeing that online access to mental health clinicians’ written notes could enhance patients’ understanding about their diagnosis, care plan, and rationale for treatments, and that access could strengthen patient recall and sense of empowerment. There was consensus that blocking mental health notes could lead to greater harm including increased feelings of stigmatisation. Yet, in contrast, surveyed experts forecast there could be an increase in patients demanding changes to their clinical notes, and that mental health clinicians would be less detailed, i.e. accurate, in their documentation after the implementation of PAEHR compared to before [30]. A qualitative survey based on the same experts concluded that there was a greater need for clarity about when access to mental health notes might be harmful, and policy and education among clinicians and patients with respect to access and best practice [31].

At the macro level within the European Union (EU), there is a tendency towards strengthening users’ security and privacy in information systems. Regulations such as the EU’s General Data Protection Regulation (GDPR) emphasise users’ right to be informed of data that are stored about them, and to be provided the opportunity to access those data. Healthcare is no exception. In the EU, the goal is that, by 2030, all citizens should have digital access to their health records [32]. If this ambition is to be fulfilled many countries will need to undertake implementation of PAEHR in the near future. Undoubtedly, many of the concerns and debates that took place in the Nordic countries prior to and during implementation, will therefore arise along similar themes in other countries. While other sectors within healthcare may have field specific challenges related to PAEHR (e.g. test results in oncology), MHC tend to attract the most concerns especially regarding how patients subjectively react to reading the content of their medical records and how best to balance respect for patient autonomy with the risk for potential harms from patient ORA [33].

In light of this, the overall aim of the present investigation is - within four European countries where PAEHR has been long implemented: Estonia, Finland, Norway, and Sweden - to provide a more extensive exploration of MHC patients’ experience with ORA. To achieve this, we analysed the MHC-data from our NORDeHEALTH 2022 Patient Survey [34]. The research questions are as follows: (1) What positive experiences do MHC patients have from using PAEHR?, (2) What negative experiences do MHC patients have from using PAEHR?, and (3) What experiences do MHC patients have with the security and privacy of PAEHRs?

## Methods

### The NORDeHEALTH 2022 patient survey

This study is reported following the STROBE guidelines (www.strobe-statement.org). To explore the positive aspects as well as problems and concerns related to patient ORA, as part of the NORDeHEALTH project [35] we conducted an anonymous online, convenience sample survey of patient users. The survey consisted of 45 questions (38 close-ended, 7 free-text) divided into 7 thematic sections. Questions were curated based on the research team’s previous studies [25, 36, 37]. The close-ended questions could be simple: containing all of the information within the question, e.g. *“Have you had a very positive experience with the platform?”*; or compound: asking to evaluate additional statements, e.g. *“Please indicate how much you disagree or agree with the following statements”*. The possible answers depended on the question, ranging from standard options, e.g. *“Yes/No/Don’t know”*, to 5-point Likert scales, e.g. *“Strongly disagree”* to *“Strongly agree”*. Due to technical differences between the survey systems, closed questions were optional in Finland and Estonia but were mandatory to answer in Norway and Sweden. The prototypical survey was constructed in English and then translated to the main national languages of each country: Norwegian in Norway, Swedish in Sweden, Finnish and Swedish in Finland, Estonian and Russian in Estonia. The surveys were pre-tested in each country to ensure that the wording was appropriate. For full description of survey design and development, see the NORDeHEALTH 2022 Patient Survey [34].

The survey was distributed independently in each country through a link placed on the national patient portal. In Norway, Sweden and Finland the survey was open for 3 weeks between January 23rd, 2022 and February 14th, 2022, and in Estonia for 9 weeks from January 23rd, 2022 to March 28th, 2022. The longer data collection period in Estonia was due to the relatively lower population, hence, lower absolute number of responses.

### Participants

Target participants for the survey were: users of the national patient portals who visited the national portals during the time of survey distribution; those aged 15 years or above in Sweden, Finland, and Estonia; those aged 16 years or above in Norway; and who spoke the national languages in which the survey was delivered. In total, we collected 29,334 responses as part of the NORDeHEALTH 2022 Patient Survey. We received 2,104 responses were from Estonia (7.17%), 4,719 responses from Finland (16.07%), 9,508 from Norway (32.40%), followed by 13,008 responses from Sweden (44.35%). For the present investigation, we focused only on participants who reported experience with MHC. To filter these responses, we used the multiple-choice question *“Have you been in contact with a HCP in the last two years for any of the following?”* with answer options *‘Mental health’*, *‘Cancer’*, ‘*Other health problems*’, *‘No treatment*’. Respondents who indicated that they had sought out MHC were considered MHC patients and included in this analysis (*N* = 6,157). The respondents who did not indicate having been in contact with MHC (*N* = 23,177) were excluded.

### Analysis

Data were summarised per country and for the total sample through descriptive statistics (count and percentage). Due to national differences in survey distribution and administration, and differences in the studied services, no statistical comparisons between countries were carried out. Percentages were calculated based on available data and excluded missing data. Calculations were performed using JASP v0.17.1 (Amsterdam University, Netherlands). Figures were built through Datawrapper (Datawrapper GmbH, Germany) and Draw.io (JGraph Ltd, UK).

## Results

In the whole sample, over three-quarters (76.77%) of the respondents were women (see Table 1). The category *‘Other’* had the lowest representation in Estonia (0.6%) compared to the other countries. In terms of age distribution, the largest proportion of participants fell within the 25–34 age range for Norway (27.36%), Sweden (26.19%), and Estonia (24.25%), while Finland had the highest proportion in the age category 55–64 years (26.68%). The most commonly reported education level was upper-secondary education (30.8%), accounting for a third of the sample. Full-time employment had the highest representation encompassing almost a third of the sample (34.56%), with Estonia having the highest proportion of respondents in this category (50.9%). However, only in Finland, most participants were retired (28.7%).

**Table 1:** Participant characteristics

	Estonia	Finland	Norway	Sweden	All countries
	*n* = 334	*n* = 693	*n* = 1,999	*n* = 3,131	*N* = 6,157
**Gender**, n (%)					
Woman	259 (78.25)	550 (79.59)	1,545 (77.29)	2,373 (75.79)	4,727 (76.77)
Man	70 (21.15)	121 (17.51)	424 (21.21)	701 (22.39)	1,316 (21.37)
Other	2 (0.60)	20 (2.89)	30 (1.50)	57 (1.82)	109 (1.77)
Missing data	3	2	-	-	5
**Age**, n (%)					
15–19 years	20 (5.99)	13 (1.90)	140 (7.00)	120 (3.83)	293 (4.76)
20–24 years	25 (7.49)	34 (4.96)	260 (13.01)	239 (7.63)	558 (9.07)
25–34 years	81 (24.25)	96 (13.99)	547 (27.36)	820 (26.19)	1,544 (25.11)
35–44 years	68 (20.36)	122 (17.78)	425 (21.26)	656 (20.95)	1,271 (20.67)
45–54 years	66 (19.76)	140 (20.41)	352 (17.61)	638 (20.38)	1,196 (19.45)
55–64 years	55 (16.47)	183 (26.68)	211 (10.56)	467 (14.92)	916 (14.89)
65–74 years	15 (4.49)	73 (10.64)	57 (2.85)	141 (4.50)	286 (4.65)
75–84 years	4 (1.20)	25 (3.64)	5 (0.25)	48 (1.53)	82 (1.33)
85 years or older	0	0	2 (0.10)	2 (0.06)	4 (0.07)
Missing data	0	7	-	-	7
**Other healthcare in the last 2 years**, n (%) ^a^					
Cancer	16 (4.79)	39 (5.63)	144 (5.70)	174 (5.56)	373 (6.1)
Other health problems	273 (81.74)	628 (90.62)	1,561 (78.09)	2,441 (77.96)	4,903 (80)
**Education**, n (%)					
No formal education	0	2 (0.29)	9 (0.45)	19 (0.61)	30 (0.49)
Primary education	29 (8.68)	59 (8.61)	213 (10.66)	311 (9.93)	612 (9.95)
Upper-secondary	79 (23.65)	223 (32.55)	672 (33.62)	920 (29.38)	1,894 (30.80)
Higher vocational education	54 (16.17)	109 (15.91)	173 (8.65)	464 (14.82)	800 (12.99)
Higher education: Bachelor’s	87 (26.05)	148 (21.61)	570 (28.51)	584 (18.65)	1,389 (22.59)
Higher education: Master’s	81 (24.25)	120 (17.52)	348 (17.41)	774 (24.72)	1,323 (21.52)
Higher education: Research	4 (1.20)	7 (1.02)	14 (0.70)	59 (1.88)	84 (1.37)
Other	-	17 (2.48)	-	-	17 (0.28)
Missing data	0	8	-	-	8
**Employment**, n (%)					
Full-time	170 (50.90)	164 (23.77)	637 (31.87)	1,156 (36.92)	2,127 (34.56)
Part-time	38 (11.38)	75 (10.87)	186 (9.30)	431 (13.77)	730 (11.86)
Student	25 (7.49)	64 (9.28)	312 (15.61)	380 (12.14)	781 (12.69)
Retired	20 (5.99)	198 (28.70)	49 (2.45)	283 (9.04)	550 (8.94)
Unemployed	33 (9.88)	74 (10.72)	82 (4.10)	143 (4.57)	332 (5.39)
Not able to work	33 (9.88)	80 (11.59)	528 (26.41)	397 (12.68)	1,038 (16.87)
None of the above	15 (4.49)	35 (5.07)	205 (10.26)	341 (10.89)	596 (9.68)
Missing data	0	3	-	-	3

Note: Percentages were calculated per national sample and for all countries in total. In Estonia and Finland, answering all questions was not mandatory for submission so there is missing data. All questions in Norway and Sweden were mandatory, hence no missing data. Only Finland had the answer option ‘Other’ by Education

^a^ A multiple-choice question, will not add up to 100%

**Fig. 1:**
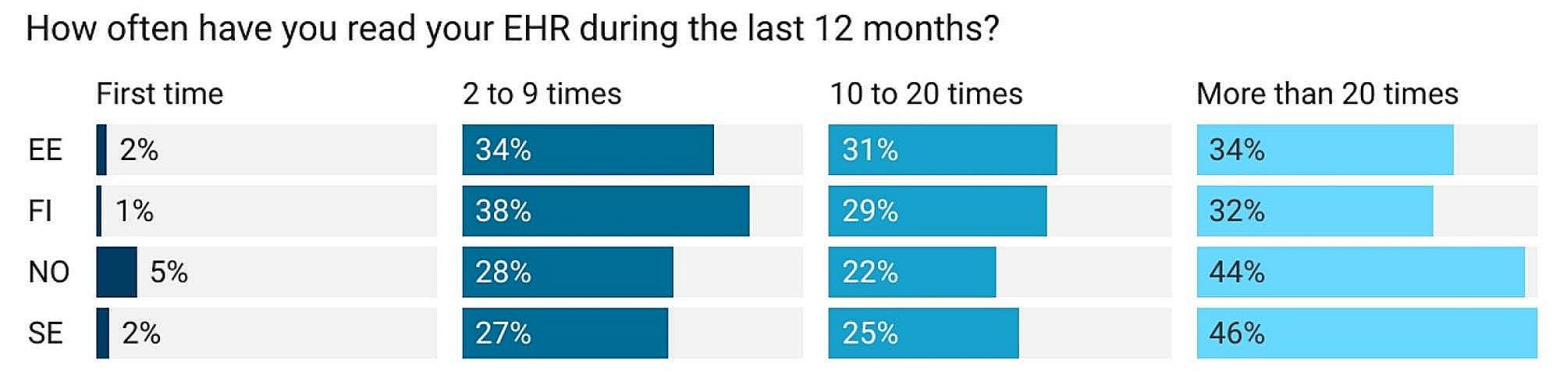
Occasions of accessing the EHR in the last 12 months Note: EE: Estonia, FI: Finland, NO: Norway, SE: Sweden

Few of the respondents were first-time users of the PAEHR services (see Fig. 1). Most respondents were returning users who reported accessing the service more than ten times during the previous twelve months, with the largest category reporting more than 20 occasions of access.

### Positive experiences with patient ORA

Almost half of all respondents reported having had a very positive experience with the health records (in total, 45%), see Table 2. Estonia had the highest proportion (59.88%) and Finland had the lowest (40.17%).

**Table 2:** Positive experiences

	Estonia	Finland	Norway	Sweden	All countries
	*n* = 334	*n* = 693	*n* = 1,999	*n* = 3,131	*N* = 6,157
**Had a very positive experience with the health record,** n (%)	200 (59.88)	276 (40.17)	986 (49.32)	1,309 (41.81)	2,771 (45.01)
Missing data	0	6	-	-	6

Note: Percentages were calculated per national sample and for all countries in total. In Estonia and Finland, answering all questions was not mandatory for submission so there are missing data. All questions in Norway and Sweden were mandatory, hence no missing data

**Fig. 2:**
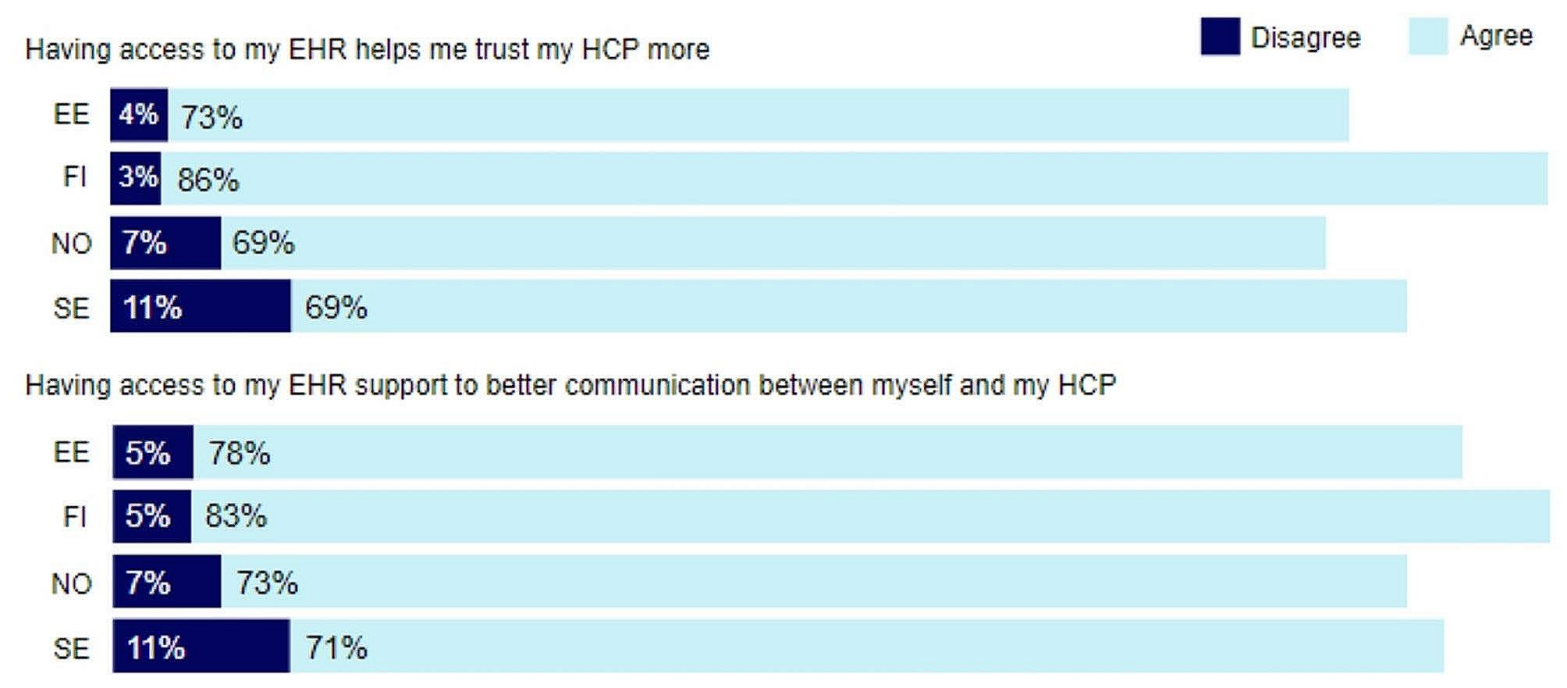
Perceived benefits from patient ORA Note: EE: Estonia, FI: Finland, NO: Norway, SE: Sweden. The label *‘Agree’* includes the Likert scale items ‘*Strongly agree’* and ‘*Somewhat agree’*, and the label *‘Disagree’* includes *‘Strongly disagree’* and *‘Somewhat Disagree’*. Neutral statements are excluded, hence, the total percentage does not add up to 100%

In all countries, the majority of respondents agreed that accessing their EHR helped them trust their HCP more (see Fig. 2). Highest agreement was in Finland (86%), while the highest number of disagreement was in Sweden (11%). Similarly, Finnish respondents (83%) had the highest agreement when considering whether access to the EHR supported the communication between themselves and their HCP, in terms of patient ORA supporting better communication with healthcare professionals, compared to the highest disagreement found in Sweden (11%).

### Negative experiences with patient ORA

Compared to the rate of positive experiences, fewer participants indicated having had a very negative experience (in total, 29.48%), see Table 3. Almost half of all respondents (47.89%) reported encountering errors in their health records, with Sweden having the highest proportion (50.65%) and Estonia the lowest (21.26%). A significant proportion of the respondents among all the countries (35.49%) discovered omissions in their health records. Overall, a third reported feeling offended while reading their health records, with Sweden having the highest proportion of offended respondents (37.78%) and Estonia the lowest (18.32%).

**Table 3:** Negative experiences

	Estonia	Finland	Norway	Sweden	All countries
	*n* = 334	*n* = 693	*n* = 1,999	*n* = 3,131	*N* = 6,157
**Had a very negative experience with the health record**, n (%) ^a^	79 (25.57)	202 (29.66)	524 (26.21)	999 (31.91)	1,804 (29.48)
Missing data	25	12	-	-	37
**Errors**, n (%)					
Yes	71 (21.26)	319 (46.23)	971 (48.57)	1,586 (50.65)	2,947 (47.89)
No	182 (54.49)	251 (36.38)	635 (31.77)	904 (28.87)	1,972 (32.04)
Don’t know/don’t remember	81 (24.25)	120 (17.39)	393 (19.66)	641 (20.47)	1,235 (20.07)
Missing data	0	3	-	-	3
**Omissions**, n (%)					
Yes	78 (28.16)	243 (35.17)	754 (37.72)	1,089 (34.78)	2,164 (35.49)
No	116 (41.88)	235 (34.01)	621 (31.07)	1,059 (33.82)	2,031 (33.31)
Don’t know/don’t remember	83 (29.96)	213 (30.82)	624 (31.22)	983 (31.40)	1,903 (31.21)
Missing data	57	2	-	-	59
**Offended**, n (%)					
Yes	61 (18.32)	175 (25.25)	724 (36.22)	1,183 (37.78)	2,143 (34.81)
No	272 (81.68)	518 (74.75)	1,275 (63.78)	1,948 (62.22)	4,013 (65.19)
Missing data	1	0	-	-	1

Note: Percentages were calculated per national sample and for all countries in total. In Estonia and Finland, answering all questions was not mandatory for submission so there is missing data. All questions in Norway and Sweden were mandatory, hence no missing data

^a^ Calculations include only data from participants who answered ‘Yes’ to the survey item, and therefore, do not add up to 100%

Participants who indicated that they had encountered an error in their records were asked a follow-up question to rate the importance of the worst mistake they had found. The results varied across the countries, see Table 4. A higher proportion of respondents in Sweden (50.13%) and Norway (44.59%) considered the worst mistake as *‘Very important’*, while Finland (11.71%) and Estonia (18.84%) had the lowest proportions in this category. Participants who indicated they had encountered an omission responded to the seriousness of the most important missing information in their records. The majority of respondents in Sweden (57.12%) considered the missing information as *‘Very serious’*, while Finland (9.5%) had the lowest proportion. Compared to Sweden, the most common response in Norway (46.68%), Finland (36.78%), and Estonia (54.55%) was that the missing information was *‘Somewhat important’*. When respondents discovered a mistake or missing information in their records, their actions varied. The most common response in all countries was *‘Did nothing’*.

**Table 4:** Perceived seriousness of errors or omission and reaction to them among respondents who encountered an error or omission

	Estonia	Finland	Norway	Sweden	All countries
**How important was the worst mistake for you?**, n (%) ^a^	*n* = 69	*n* = 316	*n* = 971	*n* = 1,586	*N =* 2,942
Very	13 (18.84)	37 (11.71)	433 (44.59)	795 (50.13)	1,278 (43.44)
Somewhat	39 (56.52)	127 (40.19)	409 (42.12)	586 (36.52)	1,161 (39.46)
Not at all	16 (23.19)	112 (35.44)	90 (9.27)	148 (9.33)	366 (12.44)
Not sure	1 (1.45)	40 (12.66)	39 (4.02)	57 (3.59)	137 (4.66)
**How serious was the most important missing information for you?**, n (%) ^b^	*n* = 77	*n* = 242	*n* = 754	*n* = 1,089	*N =* 2,162
Very	16 (20.78)	23 (9.50)	262 (34.75)	622 (57.12)	923 (42.69)
Somewhat	42 (54.55)	89 (36.78)	352 (46.68)	372 (34.16)	855 (39.55)
Not at all	10 (12.99)	76 (31.40)	46 (6.1)	20 (1.84)	152 (7.03)
Not sure	9 (11.69)	54 (22.31)	94 (12.47)	75 (6.89)	232 (10.73)
**Did you do any of the following when you found a mistake or missing information in your EHR?**, n (%) ^c^	*n* = 104	*n* = 377	*n* = 1,252	*n* = 1,918	*N =* 3,651
Did nothing	68 (65.38)	147 (38.99)	689 (55.03)	792 (41.29)	1,696 (46.45)
Contacted the healthcare unit via phone	13 (12.50)	67 (17.77)	147 (11.74)	357 (18.61)	584 (15.99)
Informed the HCP at the next visit	12 (11.54)	122 (32.36)	310 (24.76)	499 (26.02)	943 (25.83)
Something else	11 (10.58)	41 (10.88)	106 (8.47)	270 (14.08)	428 (11.72)

Note: Percentages were calculated per national sample and for all countries in total. Estonia and Finland had non-mandatory questions, hence, the data will not add up to data from the ‘Yes’-responses of error/omission

^a^ The statistics includes only data from participants who answered ‘Yes’ to the survey “Have you ever found anything in your EHR you thought was wrong (not misspellings/typographical)?”

^b^ The statistics includes only data from participants who answered ‘Yes’ to the survey “Have you ever found anything in your EHR you thought was missing?”

^c^ The statistics includes only data from participants who answered ‘Yes’ to the survey items about to have found anything wrong in the EHR and/or missing information in the EHR

### Security and privacy

In total, 3.06% of the respondents reported family, friends or others had demanded access to the health records, while 10.72% experienced that someone had seed the health records that they did not want to share with (see Table 5). Finland had the highest proportion of respondents (5.63%) who experienced demands for access to their health records by family, friends or others, while Sweden had the lowest (2.62%). In terms of privacy breaches, Finland had the highest number (15.94%) of respondents who reported someone had seen their health records without their consent, whereas Sweden had the lowest number (8.18%). There was also variation in the level of uncertainty about privacy breaches, with Sweden having the highest proportion (31.33%) of respondents who were unsure if someone had accessed their health records without consent, while Norway had the lowest (15.11%).

**Table 5:** Patients’ experiences with security and privacy

	Estonia	Finland	Norway	Sweden	All countries
	*n* = 334	*n* = 693	*n* = 1,999	*n* = 3,131	*n* = 6,157
**Experienced that family, friends, or another have demanded access to the health records**, n (%)					
Yes	11 (3.46)	39 (5.63)	56 (2.80)	82 (2.62)	188 (3.06)
No	289 (90.88)	563 (81.24)	1,892 (94.65)	2,888 (92.24)	5,632 (91.71)
Don’t know	18 (5.66)	91 (13.13)	51 (2.55)	161 (5.14)	321 (5.23)
Missing data	16	0	-	-	16
**Experienced that someone has seen the health records that you did not want to share**, n (%)					
Yes	31 (9.37)	109 (15.94)	263 (13.16)	256 (8.18)	659 (10.72)
No	242 (73.11)	456 (66.67)	1,434 (71.74)	1,894 (60.49)	4,026 (65.52)
Don’t know	58 (17.52)	119 (17.40)	302 (15.11)	981 (31.33)	1,460 (23.76)
Missing data	3	9	-	-	12

Note: Percentages were calculated per national sample and for all countries in total. In Estonia and Finland, answering all questions was not mandatory for submission so there is missing data. All questions in Norway and Sweden were mandatory, hence no missing data

**Fig. 3:**
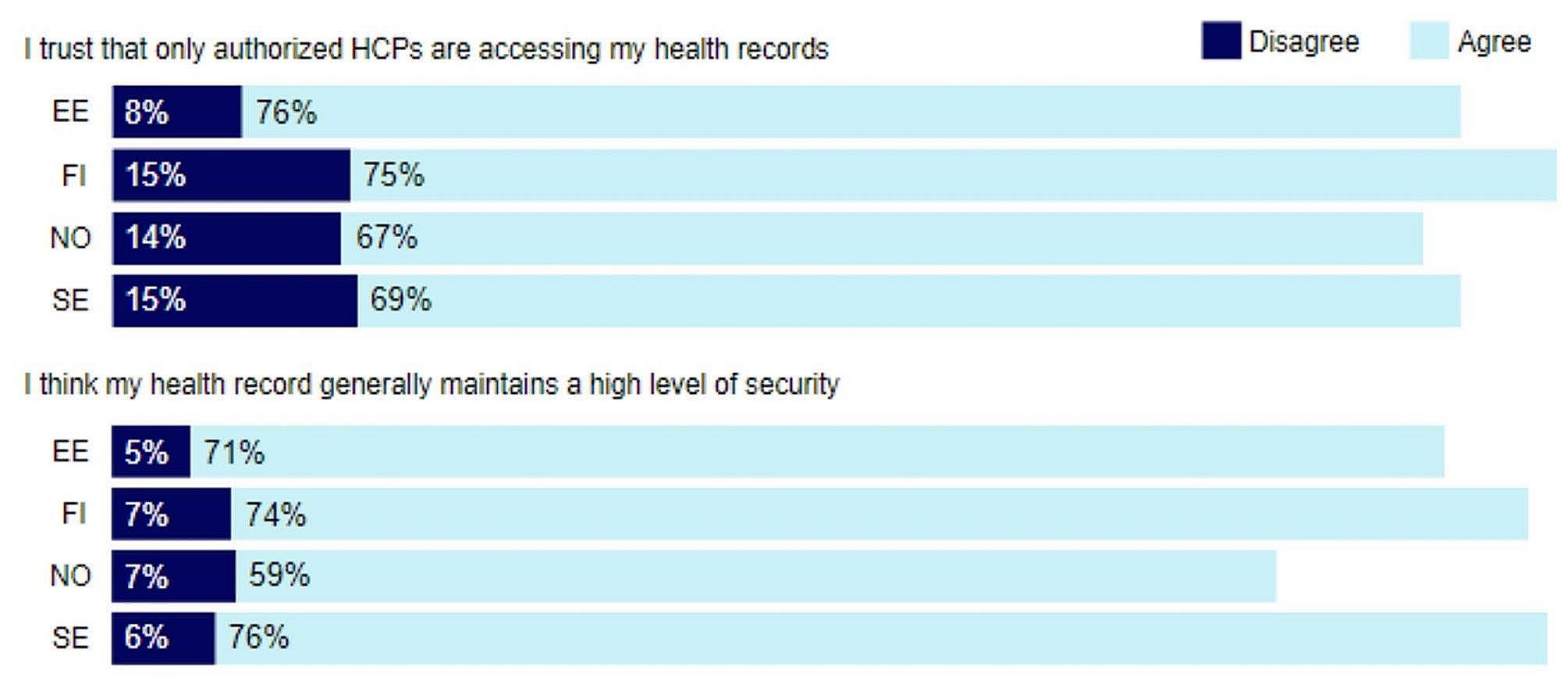
Experiences with security and privacy Note: EE: Estonia, FI: Finland, NO: Norway, SE: Sweden. The label *‘Agree’* includes the Likert scale items ‘*Strongly agree’* and ‘*Somewhat agree’*, and the label *‘Disagree’* includes *‘Strongly disagree’* and *‘Somewhat Disagree’*. Neutral statements are excluded, hence, the total percentage does not add up to 100%

The majority of respondents in all countries expressed trust in authorised access to their health records, with respondents from Estonia (76%) reporting the highest agreement, and Norway the lowest (see Fig. 3). Regarding the security of the health records, respondents from Sweden (76%) reported the highest agreement, and Norway the lowest.

## Discussion

### Main findings

The aim of the present study was to provide a more extensive exploration of MHC patients’ experiences with ORA in four European countries where PAEHRs has been long implemented. In total, a greater proportion of participants reported positive experiences with patient ORA as opposed to negative ones. This tendency was found in all four countries. Approximately one-third of the MHC respondents recounted distinctly negative experiences with the health record, the proportion ranging from 26 to 32% across countries. Notably, around one-third of the patients in MHC who participated in our study expressed feeling offended upon reviewing the contents of their health records, ranging from 18 to 38%. Half of the respondents detected errors in their medical documentation, countries ranging from 21 to 51%. In a similar fraction, one-third, reported the presence of omissions, ranging from 28 to 38%.

### Positive experiences

The majority of the respondents indicated that having access to the EHR improved their trust and communication with the HCP, echoing the results of earlier studies [38, 39]. A majority also trusted that only authorised personnel were accessing their EHR, with only 8–15% disagreeing. We did not specifically investigate the respondents’ overall satisfaction with patient ORA, but in all countries more respondents reported positive experiences than negative experiences, a tendency also found in all included studies in a recent scoping review on patients in MHC’s experiences with ORA [19], and the international Delphi study on the harms and benefits of patient access to mental health notes [30].

### Errors and omissions

In total, almost half of the respondents reported identifying errors in their documentation. Notably, the highest proportion were found in Sweden, more than twice than in Estonia. This is likely influenced by the fact that in Estonia, only epicrisis is shared with MHC patients, while a more extensive suite of documentation is provided in Sweden. In our study, we differentiated between incorrect information and missing information by asking separate questions about error and omissions. It is possible that omissions can be reported as errors and vice-versa. Consequently, it is appropriate to approach errors and omissions as related concepts, and previous qualitative studies can give an indication to drivers behind the commonly reported errors and omissions in our data. A study that included military veterans’ ORA pointed out that interviewees did not appreciate when the notes did not reflect what had occurred during the session, or they identified wrong information or outdated copy-pasted sections [39]. Similarly, in a study with PAEHR users in Finland, analysis of free text answers indicated that many users found that the notes differed from what they had experienced, while others wished for either more or less detailed notes [36]. In a qualitative study [18] among users who identified as Sámi who reported mental healthcare experience in Norway, a respondent stated that they had spent the large part of a consultation emphasising his extended family relations, but it was not reflected in the notes, and this led to disappointment for the patient. This exemplifies the possibility that similar documentation procedures might yield different appraisal from patients, depending on both individual and cultural factors.

### Action taken after noticing errors and omissions

In previous studies, HCPs have voiced concern that patient ORA might increase workload. A qualitative study suggested that HCP were concerned that patient ORA might require them to allocate time to handle phone calls, discussion with patients and amending notes [40]. In a review that summarised HCP perspectives on patient ORA in 2015 [41], half of the included studies expressed concerns that patient ORA increased HCP workload. The results from the present study suggest that patients with ORA frequently identify errors and omissions in their notes, and out of those who found errors, half of them report to have taken action following the discovery. It is likely that action from patients after reading their notes may convert to a reaction in healthcare, at least to handle the request but possibly also explaining or negotiating. Supporting this assumption, a previous study in Norway found that more than a third of HCP and administrative staff had received questions from patients or relatives related to the use of PAEHR [24]. Consequently, and in line with previous research, our results suggest that patient ORA can affect HCP workload. On the other hand, it is likely that errors in the documentation have the potential to introduce clinical errors and patient safety issues, either throughout the treatment, or afterwards. It is therefore likely that patients identifying errors in their documentation and taking action to have it explained or amended, can help avoid treatment errors. In a study among HCP in Norway after the implementation of PAEHR, 25% agreed that they noticed patients were better informed about diagnosis, treatment or follow-up than before implementation [28]. The medical record serves the purpose of a work tool for HCP, a legal documentation of clinical assessments and provided health and, after the introduction of PAHER, increasingly a source of information and empowerment for the patient.

Negotiating these sometimes conflicting requirements is not a trivial task; decisions about whether to amend perceived patient errors, or failures to modify records may lead to legal consequences that have not yet been fully explored [42]. Furthermore, sometimes HCP may make clearly relevant and decisive observations that they are mandated to document, but in doing so risk offending or even introducing conflict in the HCP-patient relationship, which when unresolved can negatively impact the treatment. The discourse on how to balance the different requirements is not settled, illustrated by the Chair of the Norwegian Psychiatric Associations recent statement that the medical record is primarily a work tool for HCP, and that ORA does not imply that the patient should be able to understand everything in it [43]. It is likely that the risk of offence or mismatch between patient expectations and documentation is to some degree a built-in price to pay for transparency, particularly in the MHC field, where the EHR usually contains substantial amounts of qualitative and emotionally loaded content. Still, it is likely that a move towards reimagining the EHR as a multi-purpose tool, and incorporating patient feedback, both to adapt in ongoing individual treatment contacts, as well as patient-informed HCP training can mitigate the dilemma between patient transparency and potential harm [33]. Similarly, HCP can be trained to document healthcare with emphatic and supportive language, and avoid clearly paternalistic, offensive or derogatory formulations [44].

### Patient offence at online health records

The present study is to our knowledge the largest study examining perceived offence among MHC users accessing their PAEHR. Only a few studies have previously asked respondents to indicate if they took offence from reading notes in MHC. Previously, a mixed-methods study used a graded response (yes, somewhat/a little, not at all, don’t know) to indicate whether readers of psychotherapy notes, isolated from general psychiatry notes, had taken offence and found that 7 out of 85 (8.2%) responded somewhat/ a little or yes to the item [29]. Similarly, a pilot study [45] that included respondents based on HCP clinical assessment found that 8.9% either agreed or strongly agreed that they felt offended when reading mental health notes. Compared to previous studies that used either isolated psychotherapy notes or included respondents based on HCP clinical assessment, our study employed a broader recruitment strategy, providing all patients that accessed the PAEHR service and had received MHC the opportunity to respond. Therefore, our sample contains users from all care levels of MHC, including primary care, outpatient specialised care, hospitalised patients and patients that have received emergency care. Thus, the sample included in the present study is likely to tap into clinical sub-populations more susceptible to experiencing offence.

Furthermore, we observed differences between the Nordic countries included in the present study, with respondents from assumed culturally similar countries (Sweden and Norway) reporting comparable levels of offence, twice the proportion of that observed in the Baltic country of Estonia. It is possible that sampling methods, cultural differences, different documentation practices, or differences in the information provided through the PAEHR affect whether patients report feeling offended. Consequently, sampling methods, clinical sub-populations and cultural differences should be taken into account when comparing the proportion of offended patients in our results with previous studies. Nevertheless, as indicated by a large thematic analysis of free-text answers from patients in a general outpatient healthcare setting [46] the feeling of being offended can be sorted into three thematic domains: errors and surprises, labelling, and disrespect. The large proportions of reported errors and omissions in our data may have constructed overlap with the concept of offence (i.e. an error or omission in the notes is also causing offence), and contribute to the large proportion of offended respondents.

### Perceptions of PAEHR’s security and privacy

Although the number of respondents who experienced breaches in the security and privacy of their records was very small in our study, it is a significant number. Between 2.5% and 6% of MHC participants reported someone demanding access to their records and between 8% and 16% reported that someone accessed against their will. Finland had the highest proportion of such incidents. The reason for this is uncertain but may be related to a recent hacking of the PAEHR service [47]. A recent qualitative study from Sweden, found that some respondents were concerned about who was able to access their medical information [48], and in the US a survey reported that 14% of the respondents were extremely concerned about privacy [29]. Another US survey found that 41% of MHC patients in primary care expressed concerns about privacy [49]. In our study we found a lower rate, potentially due to asking about personal experiences and not opinions. We did not explore these experiences further in terms of underlying causes. It is possible that severe experiences such as an MHC patient being threatened by a family member about releasing information to others, and milder instances such as an unknown name appearing in the read log listing who had accessed the document, are both included in our results. It is important to note that patient users in general are in a disadvantaged position when determining whether the security and privacy of their EHRs has been breached. This is particularly important for MHC users who experience more unwanted access than other patients [50].

### Limitations

The present study reports from the largest cross-nationally distributed survey aimed to gather experiences from PAEHR users with MHC experience. Due to the lack of demographic characteristics of the service user populations, it was not possible to estimate whether our samples are representative. Further, the tendency of respondents to provide favourable responses must be considered when employing self-reported measurements. In our study, we used an anonymous survey that were distributed digitally. This method is known to produce less social desirability bias than pan-and-paper and open-identity measurements [51]. The four participating countries, Estonia, Finland, Norway and Sweden, are similar in terms of predominantly public healthcare systems that have provided residents with patient ORA on a national level, for at least a decade. Still, country-specific implementations of patient ORA caution drawing broad conclusions about MHC users’ experiences. For example, users in Estonia could only read their epicrisis which limits the amount of content they see and thus reduces the opportunities to identify errors or omissions, or indeed taking offence. Further research is needed into analysing the relationship between available content in the records and rates of errors and offence. There were differences in survey distribution strategies. The survey was designed in English, and then translated into Norwegian, Swedish, Finnish, Estonian and Russian, possibly introducing lingual nuance differences in the distributed versions. Additionally, the way users were introduced to the survey, as well as the visual presentation varied slightly between the countries [34]. Our data trends could have been affected by whether or not the respondents accessed their EHR immediately before answering the survey, and whether or not the questions were mandatory. For example, the Estonian survey was placed externally from the PAEHR as a post alongside other news, and thus some Estonian respondents could have responded to the survey without ever reading their records. In Norway and Sweden, the questions were mandatory compared to Estonia and Finland, where the questions were optional, so respondents could choose not to respond to some questions. Finally, the medical information provided in the PAEHR service varies between countries. Due to these differences in the national ORA services, survey distribution and administration, the underlying assumptions for each sample and prerequisites for calculating weather the observed differences were due to chance were not present. Consequently, the results were presented in descriptives.

## Conclusions

While the results from the present study align with previous research, suggesting that patient ORA provides clear benefits experienced by MHC patients, we also identified some concerns. Many of our respondents perceived errors and omissions in their MHC record, or reported being offended by the record content. All four countries included in the present study have relatively mature PAEHR services that were implemented a decade ago, and most of those who responded to the survey were returning users. Thus, it would not be appropriate to attribute the observed problems and concerns to post-implementation turmoil. Future research should aim to identify the cases where changes to the clinical documentation practice could be made to avoid offending patients, starting with the changes that would clearly not compromise the EHRs’ function as a work-tool for HCP. Further, in settings where PAEHR is offered as a nation-wide service, it is likely that some sections of demographics are more likely to experience the negative aspects. A research effort to identify sub-populations that experience more problems with regards to patient ORA could help focus the guidance provided to HCPs and patients [52]. Finally, health information, particularly relating to MHC may be one of the most sensitive categories of data that is routinely stored for an individual. Although relatively seldom, patients report receiving requests to share sensitive information with their social relations. To mitigate the risk of unwanted sharing, patients could potentially be provided the opportunity to control the sections of their medical documentation that should be reachable through PAEHR.

### Electronic supplementary material

Below is the link to the electronic supplementary material.

Supplementary Material 1

## Data Availability

Data used in the present analysis may be provided upon reasonable request to Maria Hägglund (maria.hagglund@kbh.uu.se) and after approval from all data owners.

## Abbreviations

EHR: Electronic Health Record
HCP: Healthcare Professional
MHC: Mental Healthcare
ORA: Online Record Access
PAEHR: Patient-Accessible Electronic Health Record
